# Comparison of the effect of vaginal misoprostol and evening primrose oil capsule with misoprostol alone on the consequences of abortion in women with intrauterine fetal death: a randomized clinical trial

**DOI:** 10.1186/s12906-023-04082-w

**Published:** 2023-07-19

**Authors:** Hadis Hashemi, Seyedeh Batool Hasanpoor-Azghady, Masoumeh Farahani, Leila Amiri-Farahani

**Affiliations:** 1 Department of Midwifery and Reproductive, School of Nursing and Midwifery, Iran University of Medical Sciences, Tehran, Iran; 2 Department of Midwifery and Reproductive, Nursing and Midwifery Care Research Center, School of Nursing and Midwifery, Iran University of Medical Sciences, Rashid Yasemi st., Valiasr St., Tehran, 1996713883 Iran; 3 Department of Obstetrics and Gynecologists, Alborz University of Medical Sciences, Karaj, Iran

**Keywords:** Consequences of abortion, Vaginal misoprostol, Evening primrose oil, Intrauterine fetal death

## Abstract

**Background:**

Misoprostol is the choice drug for inducing an abortion with intrauterine fetal death, but it has several side effects that increase with accumulating the dose received. Induction abortion with cheap and non-invasive methods with minimal complications is essential. This study aimed to compare the effect vaginal misoprostol plus vaginal evening primrose oil capsule with vaginal misoprostol alone on the consequences of abortion in pregnant women with intrauterine fetal death at 12–20 weeks of pregnancy.

**Methods:**

This study is a randomized, triple-blind clinical trial with two parallel groups at a ratio of 1:1. We randomized 82 women with indications of termination of pregnancy due to intrauterine fetal death into two groups. The experimental group (n = 42) received 200 mcg of misoprostol tablet with 1000 mg evening primrose oil capsule intravaginal. The control group (n = 40) received 200 mcg of misoprostol tablet with 1000 mg evening primrose oil placebo capsule intravaginal. Both groups received the drugs every 4 h for up to five doses. The primary outcome was the mean induction-to-fetal expulsion interval. Secondary outcomes were the mean dose of misoprostol, the highest pain intensity in the induction-to-fetal expulsion interval, the frequency of participants requiring blood transfusion, curettage, and the frequency of side effects of misoprostol or evening primrose oil. Pain intensity was measured through the Visual Analog Scale.

**Results:**

The mean age of the experimental group was 32.30 ± 6.19 years, and the control group was 30.27 ± 7.68 years. The mean gestational age of the experimental group was 15.29 ± 2.26 weeks, and the control group was 15.10 ± 1.89 weeks. The mean induction-to-fetal expulsion interval in the experimental group (3.12 ± 2.17 h) was significantly lower than that in the control group (8.40 ± 4.1 h) (p < 0.001). The mean dose of misoprostol received in the experimental group (271.42 ± 115.39 mcg) was significantly lower than that in the control group (520 ± 201.53 mcg) (p < 0.001). Also, the mean pain intensity in the experimental group (5.02 ± 0.60) was significantly lower than that in the control group (8.65 ± 1.001) (p < 0.001). The two groups were not significantly different in the frequency of blood transfusion requirements, analgesia and drug side effects. The need for curettage in the experimental group (4.8%) was significantly lower than that in the control group (47.5%) (p < 0.001).

**Conclusions:**

Vaginal administration of evening primrose oil with misoprostol reduced duration of time of fetal expulsion, pain intensity, mean dose of misoprostol received, and the need for curettage in participants. Therefore, we suggest this method for induced abortion in women with intrauterine fetal death.

**Trial registration:**

IRCT20181207041873N3. Dated 16/2/2021 prospectively registered https://en.irct.ir/user/trial/53681/view.

## Introduction

Intrauterine fetal death (IUFD) is one of the adverse outcomes of pregnancy [1] that more than three million pregnant women face annually [2]. Fetal death is one of the indications of induced abortion [3]. Misoprostol is recommended by the World Health Organization (WHO) [4] and the International Federation of Gynecology and Obstetrics (FIGO) [5] as the drug of choice for termination of pregnancy in the first and second trimesters [3, 4]. The dose of misoprostol is determined based on fetal viability and gestational age [4, 5]. The FIGO recommended dose for termination of pregnancy due to IUFD is 200 mcg vaginally, sublingually, or buccally every four to six hours [5]. Taking misoprostol may be associated with side effects such as vomiting, diarrhea, bleeding, abdominal pain, fever, and chills; the severity and recurrence of these side effects depend on the route of administration, dose, and time interval between doses [3]. Therefore, it seems necessary to find a way to better tolerate this drug and thus make it more effective and safer [6]. Herbal medicines are non-invasive, safer and less expensive than chemical medicines [7]. According to scientific evidence, evening primrose oil (EPO) helps soften the cervix and reduce pain during gynecological procedures [8–10]. Besides, it has advantages such as easy access, low cost, better acceptance by women and fewer complications [8].

Evening primrose (Oenothera biennis) is a biennial plant of the Onagraceae family. This plant is native to North America and can grow well in other countries. The primrose seed contains 14% of the oil (EPO), of which 65–75% is linoleic acid, 7–10% gamma-linolenic acid (GLA), plus oleic, palmitic and stearic acids, and steroids campesterol and β-sitosterol. Evening primrose seeds are rich in omega-6 fatty acids [11]. This plant is generally marketed as capsules. The mechanism of EPO is to facilitate the synthesis of prostaglandin E1 with its two essential fatty acids - linoleic and gamma-linolenic acids [12]. Cervical ripening is an inflammatory process, and prostaglandins play a critical role in cervical ripening by affecting pro-inflammatory agents such as cytokines and leukocytes [13].

A systematic review and meta-analysis showed that EPO is effective for cervical ripening, reducing the rate of cesarean section and shortening the duration of labor [14]. Yet, due to the high heterogeneity of the study results, the mentioned study recommends further research based on the CONSORT statement [14]. Another systematic review and meta-analysis reported that oral administration of EPO is not effective in preparing the cervix [15]. Meanwhile, other studies have shown that vaginal administration of EPO capsules in post-term pregnancies [10, 16–18] and in women who underwent gynecological surgery [8, 9] improves Bishop score and reduces the duration of cervical ripening. Despite an extensive database search, the authors did not find a study that investigated the effect of EPO on abortion induction.

So far, no serious side effects have been observed following the consumption of EPO during pregnancy and lactation [19]. EPO is well tolerated by individuals but may cause mild side effects such as headache, loose stools, and mild gastrointestinal discomfort (belching and bloating) [20, 21].

It seems that the integration of herbal medicine into modern medicine is a suitable solution to promote community health. Thus, in this regard, the present study aimed to compare the effect vaginal misoprostol plus vaginal EPO capsule with vaginal misoprostol alone on the consequences of abortion in pregnant women with IUFD at 12–20 weeks of pregnancy.

## Methods

### Study design

This study is a superiority randomized, triple-blind clinical trial with two parallel groups at a ratio of 1:1. Participants were pregnant women at 12–20 weeks of pregnancy with indications of termination of pregnancy due to IUFD. They referred to the Department of Gynecology of Kamali Hospital affiliated with the Alborz University of Medical Sciences. We collected data from March 2021 to September 2021. The Medical Research Ethics Committee of the Iran University of Medical Sciences approved this study with code (IR.IUMS.REC.1399.1106). Participants provided informed consent to take part in the research. The present study is at the Iranian Registry of Clinical Trials (IRCT) with code number IRCT20181207041873N3 and adheres to the Consolidated Standards of Reporting Trials (CONSORT) guidelines.

### Sample size estimation

We calculated the sample size using a similar study [22] with α = 0.05 and β = 0.2. Considering the minimum clinical difference of 4 h in the mean induction-to-fetal expulsion interval (primary outcome) between the two groups, which shows a statistically significant difference, the sample size of 36 people was estimated for each group. Yet, 42 people were assigned to each group, taking into account a 15% sample drop.

### Participants

Inclusion criteria included (a) Iranian nationality; (b) ≥ 18 years of age; (c) gestational age from 12 to 20 weeks; (d) having a single fetus according to ultrasound confirmation; (e) initial Bishop score less than four; (f) body mass index (BMI) less than 30; and (g) indication of termination of pregnancy due to IUFD with confirmation in two ultrasounds.

Exclusion criteria included (a) drugs abuse; (b) smoking more than ten cigarettes a day; (c) history of cesarean section; (d) contraindications of misoprostol including ectopic pregnancy, symptoms of pelvic infection or sepsis, unstable hemodynamic status, mitral stenosis, glaucoma, asthma, and bronchitis; (e) contraindications of EPO including a history of mental illness with taking phenothiazine, history of epilepsy, schizophrenia, history of bleeding disorders and use of anticoagulants; (f) medical disorders such as heart disease, lung disease, active liver disease, acute hepatitis; (g) contraindications of misoprostol/EPO include previously known drug allergies to them, having regular uterine contractions, severe anemia, and severe vaginal bleeding.

The withdrawal criteria included (a) women with drug allergies to misoprostol or EPO; (b) women with abnormal hemodynamic status during the study; (c) Failure to expel the fetus by receiving five doses of misoprostol and 20 h passed (d) need for catheterization during the abortion process, and (e) unwillingness to continue the study.

### Randomization, concealment of allocation, and blinding

We used https://www.sealedenvelope.com for random allocation, concealment, and blinding. Someone outside the research team entered information into the site, including the number of treatment groups, block sizes (four block sizes), and the number of participants. Then the site specified a code for each sample. The same person labeled the drug packages with random codes. After sampling, an uninformed statistician of the intervention type determined which participants were in the group labeled A and which in the group labeled B by randomization list.

### Intervention

In both groups, after a physical examination by a gynecologist, a blood sample was taken for complete blood count (CBC) and blood typing (ABO Group and Rh Type). The experimental group received 200 mcg of misoprostol tablet (Cytotec, Iran Daru Pharmaceutical Company) with 1000 mg EPO capsule intravaginal every 4 h, up to five doses. The control group received 200 mcg of misoprostol tablet with 1000 mg EPO placebo capsule intravaginal every 4 h for up to five doses. EPO capsule and its placebo were Products of Barij Essence Pharmaceutical Company.

Administration of 200 mcg vaginal misoprostol every four hours to terminate a pregnancy due to IUFD was based on the FIGO protocol (5). After 20 h, if fetal expulsion did not take place, the treatment was unsuccessful. In both groups of misoprostol before insertion, for faster absorption with two drops of 0.9%, the saline solution was wet. The drugs were inserted into the posterior vaginal fornix. Participants were placed on the left side for 30 min after the insertion of the medicines in the posterior fornix. We recorded during the period of hospitalization; vital signs, vaginal bleeding, possible side effects of drugs, induction-to-fetal expulsion interval, and the interval between the fetal expulsion to the placental expulsion.

If necessary, we considered 325 mg oral acetaminophen tablets (Dr Obidi Company, Iran) in case of a fever more significant than 38 degrees and 25 mg promethazine ampoule intramuscularly (Galenus Company, Iran) in case of nausea and vomiting. In the Department of Gynecology of Kamali Hospital, breathing techniques and low back massage are routinely performed for all women undergoing induction abortion treatment who request pain relief. In the present study, the researcher who did the sampling also implemented these measures for the participants. In case of pain intolerance with these two techniques, especially in participants with excessive abdominal cramping, we considered 50 mg pethidine ampoule intramuscularly (Caspian Tamin Company, Iran). If the patients were Rh-negative, they received a 300 mcg anti-D immunoglobulin (Ig) intramuscular injection after the abortion.

After fetal expulsion, an infusion of 30 international units of oxytocin in 500 ml of 0.9% saline was administered for one hour to aid placental expulsion. If the placenta was not expelled within an hour or there was severe bleeding at any stage, the patient would be a candidate for curettage. The patient’s hemoglobin was measured at admission and six hours after the abortion. A radiologist unaware of the intervention and outside the research team performed vaginal ultrasounds for all participants with the same ultrasound machine. She checked retained products of conception (RPOC) the day after the abortion. If an ultrasound demonstrated an empty uterus, the treatment was successful. If it displayed the presence of a hyperechoic mass in the uterine cavity was considered positive ultrasound evidence of RPOC, and the treatment was unsuccessful. For participants with RPOC, the physician performed the interventions listed in Table 1. We measured pain intensity twice. The first time before the intervention at admission and the second time after fetal expulsion. The second time, the participants determined the most intensity of pain in the induction-to-fetal expulsion interval.

**Table 1:** Comparison of demographic and midwifery history information of the subjects

Variables	Experimental group (n = 42)		Control group (n = 40)		p-value
	N (%)	Mean ± SD	N (%)	Mean ± SD	
^**a**^ **Age (year)**		32.30 ± 6.19		30.27 ± 7.68	0.190
^**b**^ **Education**					
Illiterate	5 (11.9)		1 (2.5)		0.391
< High school	11 (26.2)		15 (37.5)		
High school	21 (50)		20 (50)		
Academic	5 (11.9)		4 (10)		
^**c**^ **Occupation**					0.283
Housewife	33 (78.6)		35 (87.5)		
Employed	9 (21.4)		5 (12.5)		
**BMI (kg/m2))** ^**a**^		24.43 ± 2.54		24.73 ± 227	0.570
^**a**^ **Gestational age in terms of LMP (week)**					0.694
12 to 14 weeks	15 (35.7)	15.29 ± 2.26	14 (35)	15.10 ± 1.89	
14 weeks one day to 16 weeks	11 (26.2)		12 (30)		
≥ 16	16 (38.1)		14 (35)		
^**a**^ **Gestational age in terms of ultrasound (week)**					0.642
≤ 14	14 (33.3)	16.02 ± 2.29	14 (35)	15.8 ± 2.13	
14 weeks one day to 16 weeks	9 (21.4)		10 (25)		
≥ 16	19 (45.2)		16 (40)		
^**b**^ **Number of abortions**					1
0	31 (73.8)		30 (75)		
1	8 (19)		20 (8)		
2	3 (7.2)		2 (5)		
^**b**^ **Number of deliveries**					0.178
0	10 (23.8)		17 (42.5)		
1	15 (35.7)		14 (35)		
2	13 (31)		6 (15)		
≥ 3	4 (9.5)		3 (7.5)		
^**b**^ **History of curettage**					1
Yes	1 (2.4)		1 (2.5)		
No	41 (97.6)		39 (97.5)		
^**a**^ **Hemoglobin before intervention**		11.84 ± 1.25		11.72 ± 1.09	0.665
^**b**^ **Bishop score**					0.966
0	16 (38.1)		16 (40)		
1	11 (26.2)		10 (25)		
2	11 (26.2)		9 (22.5)		
3	4 (9.5)		5 (12.5)		
^**a**^ **Pain intensity before intervention**		0.42 ± 0.80		0.67 ± 0.91	0.198

^a^Independent t-test, ^b^Fisher’s exact test, ^c^Chi-square test

### Data collection

A general information questionnaire obtained demographic and midwifery history data at visit one. The questionnaire consisted of two parts: the first was demographic information include age, education, occupation, height, and weight. The second part of midwifery history information include gestational age according to the first day of the last menstrual period (LMP), gestational age according to the first-trimester ultrasound, number of abortions, number of deliveries, and history of curettage.

### Outcome measures

The primary outcome of this study was the mean induction-to-fetal expulsion interval. Secondary outcomes included the mean dose of misoprostol received, the highest pain intensity in the induction-to-fetal expulsion interval, the frequency of participants requiring blood transfusion, curettage, and the frequency of side effects of misoprostol or EPO. We recorded all secondary outcomes in the case report form (CRF).

We measured pain intensity through the Visual Analog Scale (VAS). VAS consists of a 10-cm horizontal line grading from zero to 10. Zero typically represents ‘no pain at all’ whereas 10 signifies ‘worst pain’ imaginable. VAS is one of the most commonly used measures of pain intensity in research, and various studies repeatedly confirmed its reliability [23, 24].

### Statistical methods

We performed data analysis using SPSS software version 22. The analysis process of the present study was the per-protocol approach. Chi-square or Fisher’s exact test was used to compare qualitative variables, and an independent t-test to compare quantitative variables in the two groups. The significance level in all tests was less than 0.05.

**Fig. 1:**
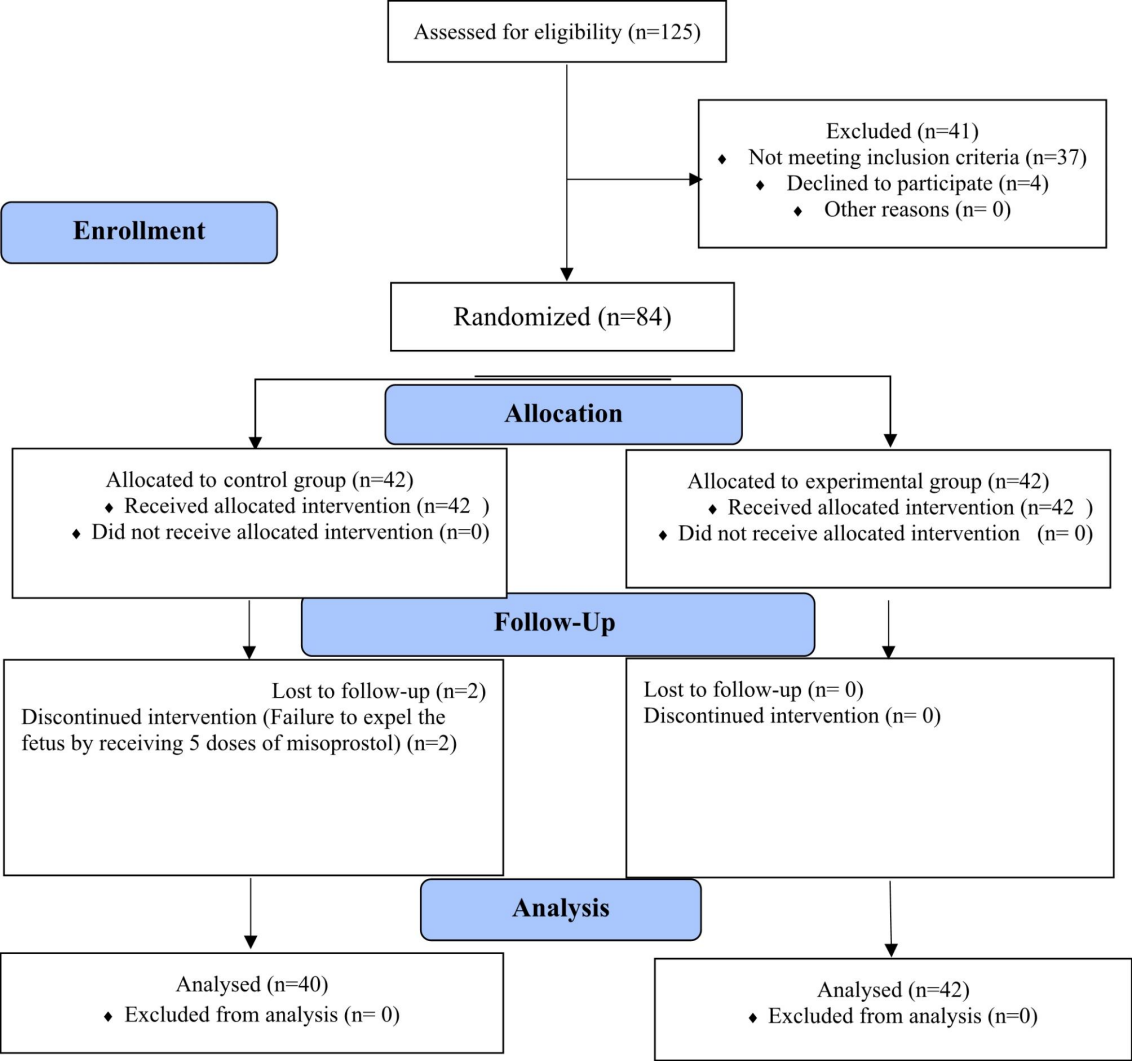
Enrolment of participants into two groups of experimental and control

## Results

Of 42 participants in each group, two people were excluded from the control group due to failure to expel the fetus after receiving five doses of misoprostol and 20 h passed. The physician applied intra-cervical Foley catheter traction to two participants. After the expulsion of the fetus, according to the ultrasound results )incomplete expulsion), both people underwent curettage. So, the experimental group consisted of 42 participants and the control group of 40 participants (Fig. 1).

The two groups did not have statistically significant differences in terms of demographic variables and history of midwifery. They were homogeneous. Demographic and midwifery history are outlined in Table 2.

Table 3 shows that the mean induction-to-fetal expulsion interval in the experimental group was significantly lower than that in the control group.

**Table 2:** Comparison of induction-to-fetal expulsion interval of experimental and control groups

Time (Hours)	Experimental group (n = 42) N (%)	Control group (n = 40) N (%)	Mean Difference	^a^ p-value	95% CI (L / H)
**≤ 4**	29 (69)	5 (12.5)		< 0.001	
4.1-8	11 (26.2)	14 (35)			
8.1–12	2 (4.8)	15 (37.5)			
12.1–16	0	4 (10)			
16.1–20	0	2 (5)			
Mean ± SD	3.12 ± 2.17	8.40 ± 4.1	5.27		3.80/6.74
range of fetus expulsion time	0.75–9.67	0.83-18			

^a^Independent t-test

The independent t-test, with p = 0.006, showed mean interval between the fetal expulsion to the placental expulsion (in minutes) in the experimental group (13.09 ± 11.74) was significantly lower than in the control group (21.7 ± 15.72).

Table 4 shows that the mean dose of misoprostol received in the experimental group was significantly lower than the control group.

**Table 3:** Comparison of the mean dose of misoprostol received by experimental and control groups

^*^Dose of misoprostol received (mcg)	Experimental group (n = 42) N (%)	Control group (n = 40) N (%)	Mean Difference	^a^ p-value	95% CI (L / H)
One dose: 200 mcg	29 (69)	5 (12.5)		< 0.001	
Two doses: 400 mcg	11 (26.2)	14 (35)			
Three doses: 600 mcg	2 (4.8)	15 (37.5)			
Four doses: 800 mcg	0	4 (10)			
Five doses: 1000 mcg	0	2 (5)			
Mean ± SD	271.42 ± 115.39	520 ± 201.53	248.57		175.59/321.54

^a^ Independent t-test

**Table 4:** Comparison of secondary outcomes of experimental and control groups

Variables	Experimental group (n = 42)		Control group (n = 40)		Mean Difference	95% CI (L / H)	p-value
	N (%)	Mean ± SD	N (%)	Mean ± SD			
^**a**^ **Highest pain intensity in the induction-to-fetal expulsion interval**		5.02 ± 0.60		8.65 ± 1.001	3.62	3.25/3.99	< 0.001
^**b**^ **Need blood transfusion**							0.105
Yes	1 (2.4)		5 12.5)				
No	41 (97.6)		35 (87.5)				
^**c**^ **Need curettage**							< 0.001
Yes	2 (4.8)		19 (47.5)				
No	40 (95.2)		21 (52.5)				
^**b**^ **Need analgesic**							0.488
Yes	0		1 (2.5)				
No	42 (100)		39 (97.5)				
^**b**^ **Fever**							0.488
Yes	0		1 (2.5)				
No	42 (100)		39 (97.5)				
^a^ **Hemoglobin six hours after the abortion**		10.86 ± 1.23		10.20 ± 1.11			0.012
^b^ **Ultrasound result after abortion**							
**Successful treatment**	36 (85.7)		11 (27.5)				< 0.001
**Unsuccessful treatment**	6 (14.3)		29 (72.5)				
^**b**^ **Intervention after the ultrasound result**							
Use of vaginal misoprostol	0		9 (22.5)				0.002
curettage	2 (4.8)		19 (47.5)				
Expectant treatment	4 (9.5)		1 (2.5)				

^a^Independent t-test, ^b^Fisher’s exact test, ^c^Chi-square test

Table 1 shows that the mean pain intensity in the induction-to-fetal expulsion interval in the experimental group was significantly lower than in the control group. Also, the two groups were not significantly different in the variables of blood transfusion requirements, analgesia and frequency of drug side effects. Only one person in the control group required analgesia following excessive abdominal cramps. She received a dose of 50 mg of intramuscular pethidine. Regarding side effects, only in the first dose of misoprostol was a case of fever in the control group with a temperature of 38.5, which received a 325 mg acetaminophen tablet. There were no other side effects (nausea, vomiting, diarrhea, chills, and rash) in any of the groups. The number of subjects requiring curettage in the experimental group was significantly less than in the control group.

## Discussion

The aim of the present study was to compare the effect of vaginal misoprostol plus EPO capsules and misoprostol alone on the consequences of abortion in women with IUFD. The results indicated that vaginal misoprostol plus EPO capsules decreased induction-to-fetal expulsion interval, pain intensity, mean dose of misoprostol received and the need for curettage in the participants. There was no significant difference between the experimental and control groups in variables of blood transfusion, analgesia and side effects of drugs.

We found no studies that examined the effects of EPO alone or plus misoprostol on abortion, so we compared our findings with studies examining the effects of EOP in other gynecological and obstetric settings (procedures).

Shah Ali et al. conducted a study to determine the effect of vaginal EPO capsules on the cervical ripening of primiparous women with post-term pregnancies. They reported that the duration of the active phase was not statistically significant in the intervention and control groups. However, the duration of the latent phase in the intervention group decreased, and the Bishop score increased [12]. In this regard, Bahmani et al.’s study also showed that vaginal EPO capsule plus misoprostol is more effective than sublingual misoprostol in cervical preparation [18]. Another study compared the vaginal administration of EPO capsules with misoprostol to dilate the cervix before gynecological procedures; it reported that cervical dilatation in the EPO group reached an appropriate level in a shorter time than in the misoprostol group [8]. Some researchers also confirmed the effectiveness of vaginal EPO capsules in softening the cervix before and during hysterosalpingography [9, 10].

Regarding the effect of EPO capsules on pain intensity, several studies with different research communities have confirmed the results of our study. In a study to determine the effect of EPO on cervical dilatation and pain intensity before and during hysterosalpingography, the intervention group reported less pain intensity than the control group when using a cervical tenaculum and injecting contrast material [25]. Also, studies have indicated the effect of EPO on reducing post-appendectomy pain [26], neuropathic pain in type 2 diabetic patients [27], and mastalgia [28, 29]. On the other hand, a study aimed at determining the effect of vaginal EPO administration on cervical ripening in primiparous women with post-term pregnancies showed that the average pain intensity in the control and intervention groups did not have a statistically significant difference [12], which was not consistent with the results of our study.

The present study showed that the mean dose of misoprostol received in the experimental group was significantly lower than the control group. Since no study was to compare with our findings, we performed the analysis based on the mechanism of misoprostol and EPO. EPO contains the effects of prostaglandins. Prostaglandins play a vital role in the cervical preparation process [12]. The mechanism of EPO is that its two essential fatty acids, linoleic and gamma-linolenic acids, help the synthesis of prostaglandin E1 [11]. Misoprostol also is an analogue of 15-methyl prostaglandin E1, which causes uterine muscle contraction and cervical dilation [4]. Therefore, it seems logical that the vaginal administration of EPO capsules can help reduce the dose of misoprostol. In this regard, Najafi et al. showed that vaginal administration of EPO capsules reduced the need for labor induction in the intervention group compared to the control group [30]. Also, the findings of another study showed that the use of vaginal EPO capsules plus misoprostol for cervical ripening in women with prolonged pregnancies is much more effective than misoprostol alone [18]. Nouri et al. reported that EPO capsules were superior to misoprostol for cervical preparation before gynecological procedures [8].

In our study, 4.8% of participants in the control group experienced side effects, while the experimental group reported no side effects. It may be due to the lower average dose of misoprostol received. In agreement with these findings, the results of the study by Khoderdoost et al., who examined the effectiveness of two doses of 200 mcg and 400 mcg of vaginal misoprostol for termination of pregnancy of 16 weeks or less, showed that side effects in the group receiving a higher dose (400 mcg) was further [31]. However, based on the results of another study that compared the effectiveness of vaginal and oral doses of 200 mcg misoprostol every six hours for induction of labor in women with IUFD, 20% of vaginal misoprostol users reported vomiting, diarrhea, and chills [32], which was higher compared to the results of our study (4.8%). Perhaps one of the reasons for this difference is the higher gestational age (above 20 weeks) in the mentioned study.

Consistent with our findings, Dickinson and Evans et al. reported that 200 mcg of vaginal misoprostol for therapeutic abortion has few side effects [33]. Also, Rabiei et al. reported 12.5% side effects such as fever, nausea, vomiting, diarrhea, and severe pain after using 200 mcg of vaginal misoprostol for therapeutic abortion [34].

Nikoei Qazvini et al. prescribed 200 mcg vaginal misoprostol every 6 h for termination of pregnancy in the second trimester of the pregnancy. 46% of participants reported adverse effects of misoprostol [35]. The difference in the results of our study and these studies regarding side effects may be due to the use of EPO capsules in the present study, which reduced the average dose received in the experimental group. Also, more frequent administration of misoprostol in the study of Nikoei Qazvini et al. (up to 48 h, which increased the duration of treatment and the number of doses received) compared to the present study (up to 20 h) may be another reason for this difference.

In two studies that examined the effect of vaginal EPO on the cervical ripening in primiparous women, the intervention and control groups were not significantly different in terms of bleeding [12, 36]. This finding was consistent with the present study. Also, Nouri et al.’s study, which examined the effect of EPO versus misoprostol for dilating the cervix before gynecological surgery, showed a statistically significant difference between the two groups in terms of side effects, so that in the misoprostol group, three cases of severe abdominal pain, two cases of diarrhea, and one case of fever reported, while no complications were observed in the EPO capsule group [8].

One of the negative consequences of misoprostol is the need for curettage following initial treatment failure and tissue retention [37]. Our study showed that 14.3% of women in the intervention group had incomplete expulsion, of which 4.8% needed curettage, while in the control group, 72.5% had incomplete expulsion and 47.5% needed curettage. However, another study in which vaginal misoprostol 200 mcg every four hours for up to four doses was used to treat missed abortions showed that 56% of participants had incomplete expulsion. But the proportion of those who needed curettage was not reported [38].

On the other hand, Ghasemi et al.’s study comparing two doses of 400 and 600 mcg of misoprostol for abortion in the second trimester did not show a significant difference in the side effects of these two regimens, although some side effects such as uterine rupture, the duration of fetal expulsion and the need to curettage was lower in the 400 mcg regimen [39]. Probably the reason for the difference between the results of the present study and the aforementioned studies is the use of EPO vaginal capsules with misoprostol, which has reduced incomplete expulsion and the need for curettage.

### Research limitations

- Light vaginal bleeding and rupture of membranes, one or both of these affect the duration of induction-to-delivery. These two variables show signs of spontaneous abortion soon. Few of our participants had mild bleeding, but we did not examine the number of people with light vaginal bleeding and rupture of membranes in the current study. We recommended that researchers pay attention to these two variables in future studies.
- We did not find a study examining the effects of EPO on abortion, so we compared our findings with studies examining the effects of EOP in other gynecological and obstetric settings.

## Conclusion

The results showed that the regimen of EPO capsule with vaginal misoprostol reduced fetal expulsion time, pain intensity, mean dose of misoprostol and the need for curettage in the experimental group. There was no significant difference between the experimental and control groups in variables of the need for blood transfusion, analgesia and side effects of drugs. Further studies are needed on women of different gestational ages and termination of pregnancy with other reasons to validate or confirm such results.

## Data Availability

The datasets used and analyzed during the current study are available from the corresponding author on reasonable request.

## Abbreviations

IUFD: Intrauterine Fetal Death
EPO: Evening Primrose Oil
VAS: Visual Analog Scale
IRCT: Iranian Registry of Clinical Trials
WHO: World Health Organization
FIGO: Fédération Internationale de Gynécologie et d’Obstétrique
GLA: Gamma-Linolenic Acid
CONSORT: Consolidated Standards of Reporting Trials
BMI: Body Mass Index
CBC: Complete Blood Count
RPOC: Retained Products Of Conception
LMP: Last Menstrual Period
CRF: Case Report Form
NVD: Normal Vaginal Delivery
